# Applying Discrete Homotopy Analysis Method for Solving Fractional Partial Differential Equations

**DOI:** 10.3390/e20050332

**Published:** 2018-05-01

**Authors:** Figen Özpınar

**Affiliations:** Bolvadin Vocational School, Afyon Kocatepe University, 03300 Afyonkarahisar, Turkey; fozpinar@aku.edu.tr; Tel.: +90-272-612-6353

**Keywords:** discrete homotopy analysis method, Caputo fractional derivative, fractional discrete diffusion equation, fractional discrete Schrödinger equation, fractional discrete Burgers’ equation

## Abstract

In this paper we developed a space discrete version of the homotopy analysis method (DHAM) to find the solutions of linear and nonlinear fractional partial differential equations with time derivative α (0<α≤1). The DHAM contains the auxiliary parameter ℏ, which provides a simple way to guarantee the convergence region of solution series. The efficiency and accuracy of the proposed method is demonstrated by test problems with initial conditions. The results obtained are compared with the exact solutions when α=1. It is shown they are in good agreement with each other.

## 1. Introduction

Fractional calculus has been of increasing interest to scientists and engineers, arising in mathematical physics, chemistry, modeling mechanical and electrical properties of real phenomena [1,2,3,4,5,6]. Fractional calculus has been recognized as a powerful instrument to discover the secret directions of various material and physical processes that deal with derivatives and integrals of arbitrary orders [7,8,9,10,11,12,13,14,15,16].

Various techniques have been investigated to solve partial differential equations of fractional order, such as the homotopy analysis method (HAM) [17,18,19,20,21,22], homotopy perturbation method (HPM) [23,24,25,26], Adomian decomposition method (ADM) [27,28,29], meshless method [30,31,32,33], operational matrix [34,35] and so on. In 1992, Liao introduced the homotopy analysis method, a semi-analytical method, for solving strongly nonlinear differential equations [20]. The main advantage of HAM is that it provides great freedom to choose equation type and solution expression of related linear high-order approximation equations. HAM gives rapidly convergent successive approximations of the exact solutions, if such a solution exists, otherwise approximations can be used for numerical purposes. It is an analytical approach to get the series solution of linear and nonlinear partial differential equations. Unlike the other analytical techniques, HAM is independent of small/large physical parameters. Since HAM has many advantages in comparison to other analytical methods, it is employed to solve continuous problems. Hence after the discrete ADM method [36], the discrete homotopy analysis method (DHAM) was introduced in 2010 by Zhu et al. [37]. This method can be applied to complex problems containing discontinuity in fluid characteristics and geometry of the problem. In addition, it needs little computational cost as numerical method in comparison to HAM; as an analytical approach DHAM has similar advantages to continuous HAM. By means of introducing an auxiliary parameter one can adjust and control the convergence region of the solution series. This method should be employed for solving various differential equations to highlight its high capabilities in comparison with other numerical methods.

In this study, we develop the discrete homotopy analysis method (DHAM) for the fractional discrete diffusion equation, nonlinear fractional discrete Schrödinger equation and nonlinear fractional discrete Burgers’ equation with time derivative α(0<α≤1). The approximate analytical solutions of the test problems are obtained using initial conditions. The obtained solutions are verified by comparison with exact solution when α=1.

## 2. Preliminaries and Notations

### 2.1. Fractional Analysis

**Definition** **1** ([38])**.** *A real function f(x), x>0 is said to be in space $C_{\alpha },\;\alpha \in \mathbb{R}$ if there exists a real number p>α such that $f\left(x\right)=x^{p}f_{1}\left(x\right)$ where $f_{1}\left(x\right)\in C\left[0,\infty \right)$.*

**Definition** **2** ([38])**.** *A function f(x), x>0 is said to be in space $C_{\alpha }^{m},\;m\in \mathbb{N}\cup \left\{0\right\}$ if $f^{m}\in C_{\alpha }$.*

**Definition** **3** ([5])**.** *Let $f\in C_{\alpha }$ and α≥−1, then Riemann-Liouville fractional integral of f(x,t) with respect to t of order α is denoted by $J^{\alpha }f\left(x,t\right)$ and is defined as*

$$J^{\alpha }f\left(x,t\right)=\frac{1}{\Gamma \left(\alpha \right)}\int_{0}^{t}{\left(t-\tau \right)}^{\alpha -1}f\left(x,\tau \right)d\tau ,\;t>0,\;\alpha >0.$$

*The well-known property of Riemann-Liouville operator* $J^{\alpha }$ *is*

$$J^{\alpha }t^{\gamma }=\frac{\Gamma \left(\gamma +1\right)t^{\gamma +\alpha }}{\Gamma \left(\gamma +\alpha +1\right)}.$$

**Definition** **4** ([39])**.** *Form to be the smallest integer that exceeds α>0, the Caputo fractional derivative of u(x,t) with respect to t of order α>0 is defined as*

$$D_{t}^{\alpha }u\left(x,t\right)=\frac{\partial^{\alpha }u\left(x,t\right)}{\partial t^{\alpha }}=\{\begin{array}{cc} \frac{1}{\Gamma \left(m-\alpha \right)}\int_{0}^{t}{\left(t-\tau \right)}^{m-\alpha -1}\frac{\partial^{m}u}{\partial t^{m}}d\tau , & for\;m-1<\alpha <m\\ \frac{\partial^{m}u\left(x,t\right)}{\partial t^{m}}, & for\;\alpha =m\in \mathbb{N}. \end{array}$$

*Note that the Caputo fractional derivative is considered due to its suitable for initial conditions of the differential equations.*

*The relations between Riemann-Liouville operator and Caputo fractional differential operator are given as follows*

$$D^{\alpha }\left(J^{\alpha }f\left(x,t\right)\right)=f\left(x,t\right),\;J^{\alpha }\left(D^{\alpha }f\left(x,t\right)\right)=J^{\alpha }\left(J^{m-\alpha }f^{\left(m\right)}\left(x,t\right)\right)=J^{m}f^{\left(m\right)}\left(x,t\right)=f\left(x,t\right)-\overset{m-1}{\underset{k=0}{\sum }}f^{\left(k\right)}\left(x,0\right)\frac{t^{k}}{k!}.$$

### 2.2. Discrete Homotopy Analysis Method

Consider the following general difference equation respect to *j*

(1) $$N\left[u_{j}\left(t\right)\right]=0,\;j\in \mathbb{Z},\;t\in \mathbb{R},$$

where N is a linear or nonlinear operator, *j* and *t* denote the independent variables. Suppose that Δx=h and the function u(x,t)=u(jΔx,t) is the discrete function and denoted by $u_{j}\left(t\right)$.

For simplicity, we ignore all boundary or initial conditions, which can be treated in the similar way. Similarly to continuous HAM, we first construct the so-called zeroth-order deformation equation by means of the discrete HAM (DHAM)

(2) $$\left(1-p\right)L\left[\varphi_{j}\left(t;p\right)-u_{j,0}\left(t\right)\right]=p\hbar H_{j}\left(t\right)N\left[\varphi_{j}\left(t;p\right)\right],$$

where p∈[0,1] is an embedding parameter, ℏ≠0 is an auxiliary parameter, L is an auxiliary linear operator, $u_{j,0}\left(t\right)$ is an initial guess of $u_{j}\left(t\right),$ $H_{j}\left(t\right)$ denotes a nonzero auxiliary function, $\varphi_{j}\left(t;p\right)$ is an unknown function about *j*, *t*, *p*. It is important that one has great freedom to choose auxiliary things in (2). Obviously, when p=0 and p=1, it holds

$$\varphi_{j}\left(t;0\right)=u_{j,0}\left(t\right)=u_{j}\left(0\right)\;\mathrm{and}\;\varphi_{j}\left(t;1\right)=u_{j}\left(t\right),$$

respectively. Thus, as *p* increases from 0 to 1, the solution $\varphi_{j}\left(t;p\right)$ varies from initial guess $u_{j,0}\left(t\right)$ to the solution $u_{j}\left(t\right)$. Expanding $\varphi_{j}\left(t;p\right)$ in Taylor series with respect to *p*, we have

(3) $$\varphi_{j}\left(t;p\right)=u_{j,0}\left(t\right)+\overset{+\infty }{\underset{m=1}{\sum }}u_{j,m}\left(t\right)p^{m},\;\left(2\right)$$

where

(4) $$u_{j,m}\left(t\right)={\frac{1}{m!}\frac{\partial^{m}\varphi_{j}\left(t;p\right)}{\partial p^{m}}|}_{p=0}$$

Similarly continuous HAM by Liao [21], if the auxiliary linear operator, the initial guess, the auxiliary parameter ℏ, and the auxiliary function are so properly chosen, the series (3) converges at p=1, then we have

(5) $$u_{j}\left(t\right)=u_{j,0}\left(t\right)+\overset{+\infty }{\underset{m=1}{\sum }}u_{j,m}\left(t\right).$$

As ℏ=−1 and $H_{j}\left(t\right)=1,$ Equation (2) becomes

$$\left(1-p\right)L\left[\varphi_{j}\left(t;p\right)-u_{j,0}\left(t\right)\right]+pN\left[\varphi_{j}\left(t;p\right)\right]=0;$$

which is used in the discrete homotopy perturbation method (DHPM) [16], where as the solution obtained directly, without using Taylor series.

According to Equation (4), the governing equation can be deduced from the zeroth-order deformation Equation (DHAM). Define the vector

$${\vec{u}}_{n}=\left\{u_{j,0}\left(t\right),u_{j,1}\left(t\right),\cdots u_{j,n}\left(t\right)\right\}.$$

Differentiating the zeroth-order deformation Equation (2) *m* times with respect to the embedding parameter *p* and then setting *p* = 0 and finally dividing them by *m*!, we obtain the following mth-order deformation equation:

(6) $$L\left[u_{j,m}\left(t\right)-X_{m}u_{j,m-1}\left(t\right)\right]=\hbar H_{j}\left(t\right)R_{m}\left[{\vec{u}}_{m-1}\right],$$

where

(7) $$R_{m}\left[{\vec{u}}_{m-1}\right]={\frac{1}{\left(m-1\right)!}\frac{\partial^{m-1}N\left[\varphi_{j}\left(t;p\right)\right]}{\partial p^{m-1}}|}_{p=0}$$

and

$$X_{m}=\{\begin{array}{c} 0,\;m\leq 1,\\ 1,\;m>1. \end{array}$$

It should be emphasized that it is very important to ensure that Equation (3) converges at p=1 otherwise, the Equation (5) has no meaning.

**Theorem 1** (Convergence Theorem)**.** *As long as the series (5) is convergent, where $u_{j,m}\left(t\right)$ is governed by the high deformation Equation (6). It must be the solution of the original Equation (1).*

**Proof.**  If the series $\sum_{m=0}^{+\infty }u_{j,m}\left(t\right)$ is convergent, we can write

(8) $$s_{j}\left(t\right)=u_{j,0}\left(t\right)+\overset{+\infty }{\underset{m=1}{\sum }}u_{j,m}\left(t\right),$$

and it holds

$$\underset{n\to \infty }{\lim}u_{j,n}\left(t\right)=0.$$

From the mth-order deformation Equation (6) and by using the definition of $X_{m,}$ it yields

(9) $$\begin{array}{cc} \overset{+\infty }{\underset{m=1}{\sum }}\hbar H_{j}\left(t\right)R_{m}\left[{\vec{u}}_{m-1}\right] & =\overset{+\infty }{\underset{m=1}{\sum }}L\left[u_{j,m}\left(t\right)-X_{m}u_{j,m-1}\left(t\right)\right]\\ & =\underset{n\to \infty }{\lim}\overset{n}{\underset{m=1}{\sum }}L\left[u_{j,m}\left(t\right)-X_{m}u_{j,m-1}\left(t\right)\right]\\ & =L\left[\underset{n\to \infty }{\lim}\overset{n}{\underset{m=1}{\sum }}\left(u_{j,m}\left(t\right)-X_{m}u_{j,m-1}\left(t\right)\right)\right]\\ & =L[\underset{n\to \infty }{\lim}(u_{j,1}\left(t\right)+(u_{j,2}\left(t\right)-u_{j,1}\left(t\right))+(u_{j,3}\left(t\right)-u_{j,2}\left(t\right))+\cdots \\ & \;+(u_{j,n}\left(t\right)-u_{j,n-1}\left(t\right)))]\\ & =L\left[\underset{n\to \infty }{\lim}u_{j,n}\left(t\right)\right]=0. \end{array}$$

Since $\hbar \neq 0,\;H_{j}\left(t\right)\neq 0,$ then

(10) $$\overset{+\infty }{\underset{m=1}{\sum }}R_{m}\left[{\vec{u}}_{m-1}\right]=0.$$

On the other side, according to the definition (7), we have

(11) $$\overset{+\infty }{\underset{m=1}{\sum }}R_{m}\left[{\vec{u}}_{m-1}\right]=\overset{+\infty }{\underset{m=1}{\sum }}\frac{1}{\left(m-1\right)!}{\frac{\partial^{m-1}N\left[\varphi_{j}\left(t;p\right)\right]}{\partial p^{m-1}}|}_{p=0}=\overset{+\infty }{\underset{m=0}{\sum }}\frac{1}{m!}{\frac{\partial^{m}N\left[\varphi_{j}\left(t;p\right)\right]}{\partial p^{m}}|}_{p=0}=0.$$

In general, $\varphi_{j}\left(t;p\right)$ doesn’t satisfy the original Equation (1). Let

$$\varepsilon_{j}\left(t;p\right)=N\left[\varphi_{j}\left(t;p\right)\right]$$

denote the residual error of Equation (1). Obviously,

$$\varepsilon_{j}\left(t;p\right)=0$$

corresponds to the exact solution of the Equation (1).

According to the above definition, the Maclaurin series of the residual error $\varepsilon_{j}\left(t;p\right)$ with respect to the embedding parameter *p* is

$$\varepsilon_{j}\left(t;p\right)=\overset{+\infty }{\underset{m=0}{\sum }}\left({\frac{1}{m!}\frac{\partial^{m}\varepsilon_{j}\left(t;p\right)}{\partial p^{m}}|}_{p=0}\right)p^{m}=\overset{+\infty }{\underset{m=0}{\sum }}\left({\frac{1}{m!}\frac{\partial^{m}N\left[\varphi_{j}\left(t;p\right)\right]}{\partial p^{m}}|}_{p=0}\right)p^{m}.$$

when p=1, the above expression gives

(12) $$\varepsilon_{j}\left(t;1\right)=\overset{+\infty }{\underset{m=0}{\sum }}{\frac{1}{m!}\frac{\partial^{m}N\left[\varphi_{j}\left(t;p\right)\right]}{\partial p^{m}}|}_{p=0}=0.$$

That is, according to the definition of $\varepsilon_{j}\left(t;p\right)$ we have the exact solution of the original Equation (1) when p=1. Thus as long as the series

$$u_{j,0}\left(t\right)+\overset{+\infty }{\underset{m=1}{\sum }}u_{j,m}\left(t\right)$$

is convergent, it must be the solution of the original Equation (1). ☐

## 3. Examples

**Example** **1.** *Consider the time fractional discrete diffusion equation*

(13) $$D_{t}^{\alpha }u_{j}\left(t\right)=D_{h}^{2}u_{j}\left(t\right)+jhD_{h}u_{j}\left(t\right)+u_{j}\left(t\right),\;0<\alpha \leq 1$$

*with initial condition*

(14) $$u_{j}\left(0\right)=jh.$$

*Discrete diffusion equation is widely used in applied sciences. For example, population growth modeled by geographical spread [40], to model ionic diffusion on a lattice [41]*, and so on. *Moreover*, *the entropy production was calculated for fractional diffusion Equation [42,43].*

*The standard central differences $D_{h}u_{j}\left(t\right)$ and $D_{h}^{2}u_{j}\left(t\right)$ are defined by*

$$D_{h}u_{j}\left(t\right)=\frac{u_{j+1}\left(t\right)-u_{j-1}\left(t\right)}{2h},\;D_{h}^{2}u_{j}\left(t\right)=\frac{u_{j+1}\left(t\right)-2u_{j}\left(t\right)+u_{j-1}\left(t\right)}{h^{2}}.$$

*Initial value problem (13) and (14) is the discrete form of initial value problem for diffusion equation*

$$D_{t}^{\alpha }u\left(x,t\right)=u_{xx}\left(x,t\right)+xu_{x}\left(x,t\right)+u\left(x,t\right),\;0<\alpha \leq 1$$

*with initial condition*

u(x,0)=x,

*where*$D_{t}^{\alpha }u\left(x,t\right)$*is Caputo fractional derivative of order*α.

*To solve Equation* (13) *by DHAM let us consider the following linear operator:*

(15) $$L\left[\varphi_{j}\left(t;p\right)\right]=D_{t}^{\alpha }\left[\varphi_{j}\left(t;p\right)\right]=\frac{\partial^{\alpha }\varphi_{j}\left(t;p\right)}{\partial t^{\alpha }}$$

*with the property that*

L[c]=0,

*where c is constant coefficients. We define the nonlinear operator as*

(16) $$N\left[\varphi_{j}\left(t;p\right)\right]=D_{t}^{\alpha }\varphi_{j}\left(t;p\right)-D_{h}^{2}\varphi_{j}\left(t;p\right)-jhD_{h}\varphi_{j}\left(t;p\right)-\varphi_{j}\left(t;p\right).$$

*Using the above definition, we construct the zeroth-order deformation equation by Equation* (2).

*It is obvious that when the embedding parameter*p=0*and*p=1*, Equation* (2) *becomes*

$$\varphi_{j}\left(t;0\right)=u_{j,0}\left(t\right)=u_{j}\left(0\right),\;\varphi_{j}\left(t;1\right)=u_{j}\left(t\right),$$

*respectively. Then we obtain the mth-order deformation equation for* m≥1 *with*

(17) $$L\left[u_{j,m}\left(t\right)-X_{m}u_{j,m-1}\left(t\right)\right]=\hbar H_{j}\left(t\right)R_{m}\left[{\vec{u}}_{m-1}\right]\Rightarrow \;u_{j,m}\left(t\right)=X_{m}u_{j,m-1}\left(t\right)+\hbar J^{\alpha }\left[H_{j}\left(t\right)R_{m}\left[{\vec{u}}_{m-1}\right]\right],$$

*where*

(18) $$R_{m}\left[{\vec{u}}_{m-1}\right]={\frac{1}{\left(m-1\right)!}\frac{\partial^{m-1}N\left[\varphi_{j}\left(t;p\right)\right]}{\partial p^{m-1}}|}_{p=0}\;\;=D_{t}^{\alpha }u_{j,m-1}\left(t\right)-D_{h}^{2}u_{j,m-1}\left(t\right)-jhD_{h}u_{j,m-1}\left(t\right)-u_{j,m-1}\left(t\right).$$

*For simplicity, we select*$H_{j}\left(t\right)=1$*in this problem. So, the approximations of*$u_{j}\left(t\right)$*are only depend on auxiliary parameter*ℏ.

*Solve the above equation under the initial condition*

$$u_{j,0}\left(t\right)=u_{j}\left(0\right)=jh$$

*we get*

$$u_{j,1}\left(t\right)=-jh\frac{2t^{\alpha }}{\Gamma \left(\alpha +1\right)}\hbar$$

$$u_{j,2}\left(t\right)=-jh\frac{2t^{\alpha }}{\Gamma \left(\alpha +1\right)}\hbar \left(\hbar +1\right)+jh\frac{2^{2}t^{2\alpha }}{\Gamma \left(2\alpha +1\right)}\hbar^{2}$$

$$u_{j,3}\left(t\right)=-jh\frac{2t^{\alpha }}{\Gamma \left(\alpha +1\right)}\hbar {\left(\hbar +1\right)}^{2}+jh\frac{2^{2}t^{2\alpha }}{\Gamma \left(2\alpha +1\right)}2\hbar^{2}\left(\hbar +1\right)-jh\frac{2^{3}t^{3\alpha }}{\Gamma \left(3\alpha +1\right)}\hbar^{3}$$

$$u_{j,4}\left(t\right)=-jh\frac{2t^{\alpha }}{\Gamma \left(\alpha +1\right)}\hbar {\left(\hbar +1\right)}^{3}+jh\frac{2^{2}t^{2\alpha }}{\Gamma \left(2\alpha +1\right)}3\hbar^{2}{\left(\hbar +1\right)}^{2}-jh\frac{2^{3}t^{3\alpha }}{\Gamma \left(3\alpha +1\right)}3\hbar^{3}\left(\hbar +1\right)+jh\frac{2^{4}t^{4\alpha }}{\Gamma \left(4\alpha +1\right)}\hbar^{4}$$

*Thus the rest of components* $u_{n},\;n>4$ *of the DHAM can be completely obtained. So, we approximate the analytical solution*

$$\begin{array}{cc} u_{j}\left(t\right) & =u_{j,0}\left(t\right)+u_{j,1}\left(t\right)+u_{j,2}\left(t\right)+u_{j,3}\left(t\right)+u_{j,4}\left(t\right)+\cdots \\ & =jh-jh\frac{2t^{\alpha }}{\Gamma \left(\alpha +1\right)}\hbar -jh\frac{2t^{\alpha }}{\Gamma \left(\alpha +1\right)}\hbar \left(\hbar +1\right)+jh\frac{2^{2}t^{2\alpha }}{\Gamma \left(2\alpha +1\right)}\hbar^{2}\\ & -jh\frac{2t^{\alpha }}{\Gamma \left(\alpha +1\right)}\hbar {\left(\hbar +1\right)}^{2}+jh\frac{2^{2}t^{2\alpha }}{\Gamma \left(2\alpha +1\right)}2\hbar^{2}\left(\hbar +1\right)-jh\frac{2^{3}t^{3\alpha }}{\Gamma \left(3\alpha +1\right)}\hbar^{3}\\ & -jh\frac{2t^{\alpha }}{\Gamma \left(\alpha +1\right)}\hbar {\left(\hbar +1\right)}^{3}+jh\frac{2^{2}t^{2\alpha }}{\Gamma \left(2\alpha +1\right)}3\hbar^{2}{\left(\hbar +1\right)}^{2}-jh\frac{2^{3}t^{3\alpha }}{\Gamma \left(3\alpha +1\right)}3\hbar^{3}\left(\hbar +1\right)\\ & \;+jh\frac{2^{4}t^{4\alpha }}{\Gamma \left(4\alpha +1\right)}\hbar^{4}+\cdots \end{array}$$

*Setting*ℏ=−1*, we get an accurate approximation solution in the following form:*

$$u_{j}\left(t\right)=jh+jh\frac{2t^{\alpha }}{\Gamma \left(\alpha +1\right)}+jh\frac{2^{2}t^{2\alpha }}{\Gamma \left(2\alpha +1\right)}+jh\frac{2^{3}t^{3\alpha }}{\Gamma \left(3\alpha +1\right)}+jh\frac{2^{4}t^{4\alpha }}{\Gamma \left(4\alpha +1\right)}+\cdots$$

$$u_{j}\left(t\right)=\overset{\infty }{\underset{n=0}{\sum }}\left(jh\right)\frac{2^{n}t^{n\alpha }}{\Gamma \left(n\alpha +1\right)}=\left(jh\right)E_{\alpha }\left(2t^{\alpha }\right),$$

*where*$E_{\alpha }$*is Mittag-Leffler function*.

$$u\left(x,t\right)=xE_{\alpha }\left(2t^{\alpha }\right)$$

*is the exact solution of the continuous form.*

**Figure 1:**
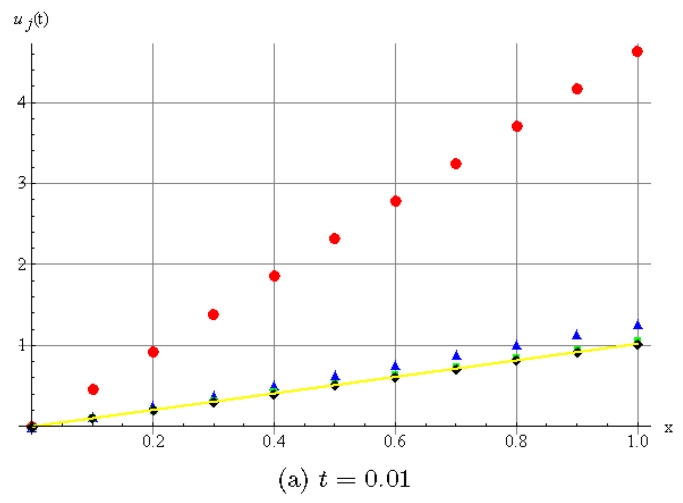

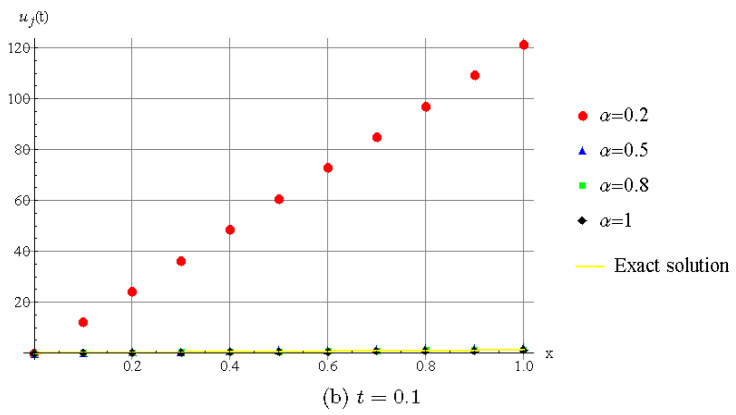
Numerical illustration of approximation solution *u*(*x*,*t*) by discrete homotopy analysis method (DHAM). (**a**) For *t* = 0.01; (**b**) For *t* = 0.1.

Figure 1 shows the DHAM approximate solution of u(x,t) for different values of α.

We can see that different fractional order lead to different diffusion behaviors.

**Figure 2:**
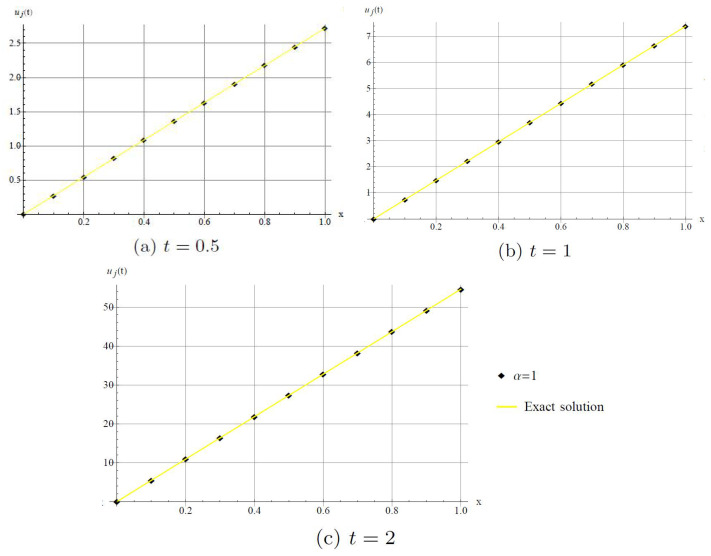
Comparison with numerical solution of *u*(*x*,*t*) by DHAM and the exact solution when α=1. (**a**) For *t* = 0.5; (**b**) For *t* = 1; (**c**) For *t* = 2.

In Figure 2, we show that the method has good agreement with the exact solution when α=1.

**Example** **2.** *Consider the nonlinear fractional discrete Schrödinger equation*

(19) $$iD_{t}^{\alpha }u_{j}\left(t\right)+D_{h}^{2}u_{j}\left(t\right)+q{\left|u_{j}\left(t\right)\right|}^{2}u_{j}\left(t\right)=0,\;j\in \mathbb{Z},\;t>0,\;0<\alpha \leq 1,$$

*with initial condition*

(20) $$u_{j}\left(0\right)=e^{ijkh}.$$

*Discrete nonlinear Schrödinger equation is widely used in applied sciences. Describing the propagation of electromagnetic waves in glass fibers, one–dimensional arrays of coupled optical waveguides [18] and light–induced photonic crystal lattices [44]. Moreover, they are an established model for optical pulse propagation in various doped fibers [45,46].*

*Discrete nonlinear Schrödinger equations are also called lattice nonlinear Schrödinger equations [47].*

*The parameter* $\varepsilon =h^{-2}$ *is called (discrete) dispersion and the parameter q is called anharmonicity.*

*Initial value problem* (19) and (20) *is the discrete form of initial value problem for Schrödinger equation*

$$iD_{t}^{\alpha }u\left(x,t\right)+u_{xx}\left(x,t\right)+q{\left|u\left(x,t\right)\right|}^{2}u\left(x,t\right)=0,\;t>0,\;0<\alpha \leq 1,$$

*with initial condition*

$$u\left(x,0\right)=e^{ikx}.$$

*we set* ${\left|u_{j}\left(t\right)\right|}^{2}u_{j}\left(t\right)=u_{j}^{2}\left(t\right){\bar{u}}_{j}\left(t\right)$.

*By means of DHAM, we choose the linear operator:*

(21) $$L\left[\varphi_{j}\left(t;p\right)\right]=D_{t}^{\alpha }\left[\varphi_{j}\left(t;p\right)\right]=\frac{\partial^{\alpha }\varphi_{j}\left(t;p\right)}{\partial t^{\alpha }}$$

*with property*L[c]=0,*where c is constant. We define the nonlinear operator as*

(22) $$N\left[\varphi_{j}\left(t;p\right)\right]=D_{t}^{\alpha }\varphi_{j}\left(t;p\right)-iD_{h}^{2}\varphi_{j}\left(t;p\right)-iq\varphi_{j}^{2}\left(t;p\right){\bar{\varphi }}_{j}\left(t;p\right).$$

*we construct the zeroth-order deformation equation by Equation* (2).

*For*p=0*and*p=1*, we can write*

$$\varphi_{j}\left(t;0\right)=u_{j,0}\left(t\right)=u_{j}\left(0\right),\;\varphi_{j}\left(t;1\right)=u_{j}\left(t\right),$$

*respectively. Thus, we obtain the mth-order deformation equation*

(23) $$L\left[u_{j,m}\left(t\right)-X_{m}u_{j,m-1}\left(t\right)\right]=\hbar H_{j}\left(t\right)R_{m}\left[{\vec{u}}_{m-1}\right],$$

*where*

(24) $$R_{m}\left[{\vec{u}}_{m-1}\right]=D_{t}^{\alpha }u_{j,m-1}\left(t\right)-iD_{h}^{2}u_{j,m-1}\left(t\right)-iq\left[\overset{m-1}{\underset{l=0}{\sum }}\overset{m-l-1}{\underset{n=0}{\sum }}u_{j,l}\left(t\right)u_{j,n}\left(t\right){\bar{u}}_{j,m-n-l-1}\left(t\right)\right].$$

*we can select again*$H_{j}\left(t\right)=1$*. Thus, the approximations of*$u_{j}\left(t\right)$*are only depend on auxiliary parameter*ℏ.

*Therefore the solution of the mth-order deformation Equation (23) for* m≥1 *become*

(25) $$u_{j,m}\left(t\right)=X_{m}u_{j,m-1}\left(t\right)+\hbar J^{\alpha }R_{m}\left[{\vec{u}}_{m-1}\right].$$

*Substituting the initial condition (20) into the system (25), we get*

$$\begin{array}{c} u_{j,1}\left(t\right)=\frac{i\omega e^{ijkh}t^{\alpha }}{\Gamma \left(\alpha +1\right)}\hbar \\ u_{j,2}\left(t\right)=\frac{i\omega e^{ijkh}t^{\alpha }}{\Gamma \left(\alpha +1\right)}\hbar \left(\hbar +1\right)-\frac{\omega^{2}e^{ijkh}t^{2\alpha }}{\Gamma \left(2\alpha +1\right)}\hbar^{2}\\ u_{j,3}\left(t\right)=\frac{i\omega e^{ijkh}t^{\alpha }}{\Gamma \left(\alpha +1\right)}\hbar {\left(\hbar +1\right)}^{2}-\frac{2\omega^{2}e^{ijkh}t^{2\alpha }}{\Gamma \left(2\alpha +1\right)}\hbar^{2}\left(\hbar +1\right)\\ \;-\frac{i\omega^{3}e^{ijkh}t^{3\alpha }}{\Gamma \left(3\alpha +1\right)}\hbar^{3}\left[1-\frac{2q}{\omega }+\frac{q\Gamma \left(2\alpha +1\right)}{\omega {\left(\Gamma \left(\alpha +1\right)\right)}^{2}}\right]\\ \vdots \end{array}$$

*where* $\omega =\left(4/h^{2}\right)sin^{2}\left(kh/2\right)-q$ *(discrete dispersion relation).*

*Thus, we can conclude that*

$$\begin{array}{cc} u_{j}\left(t\right) & =u_{j,0}\left(t\right)+u_{j,1}\left(t\right)+u_{j,2}\left(t\right)+u_{j,3}\left(t\right)+\cdots \\ & =e^{ijkh}+\frac{i\omega e^{ijkh}t^{\alpha }}{\Gamma \left(\alpha +1\right)}\hbar +\frac{i\omega e^{ijkh}t^{\alpha }}{\Gamma \left(\alpha +1\right)}\hbar \left(\hbar +1\right)\\ & -\frac{\omega^{2}e^{ijkh}t^{2\alpha }}{\Gamma \left(2\alpha +1\right)}\hbar^{2}+\frac{i\omega e^{ijkh}t^{\alpha }}{\Gamma \left(\alpha +1\right)}\hbar {\left(\hbar +1\right)}^{2}-\frac{2\omega^{2}e^{ijkh}t^{2\alpha }}{\Gamma \left(2\alpha +1\right)}\hbar^{2}\left(\hbar +1\right)\\ & -\frac{i\omega^{3}e^{ijkh}t^{3\alpha }}{\Gamma \left(3\alpha +1\right)}\hbar^{3}\left[1-\frac{2q}{\omega }+\frac{q\Gamma \left(2\alpha +1\right)}{\omega {\left(\Gamma \left(\alpha +1\right)\right)}^{2}}\right]+\cdots \end{array}$$

*Setting* ℏ=−1 *, we get an accurate approximation solution in the following form:*

(26) $$\begin{array}{cc} u_{j}\left(t\right) & =e^{ijkh}\{1-\frac{i\omega t^{\alpha }}{\Gamma \left(\alpha +1\right)}-\frac{\omega^{2}t^{2\alpha }}{\Gamma \left(2\alpha +1\right)}\\ & \;+\frac{i\omega^{3}t^{3\alpha }}{\Gamma \left(3\alpha +1\right)}\left[1-\frac{2q}{\omega }+\frac{q\Gamma \left(2\alpha +1\right)}{\omega {\left(\Gamma \left(\alpha +1\right)\right)}^{2}}\right]+\cdots \} \end{array}$$

*For the special case* α=1, *the form Equation (26) is obtained discrete plane wave solution*

$$\begin{array}{cc} u_{j}\left(t\right) & =e^{ijkh}\left\{1-i\omega t-\frac{\omega^{2}t^{2}}{2}+\frac{i\omega^{3}t^{3}}{6}+\cdots \right\}\\ & =e^{ijkh}\{1+\frac{\left(-i\right)\omega t}{1!}+\frac{{\left(-i\right)}^{2}\omega^{2}t^{2}}{2!}+\frac{{\left(-i\right)}^{3}\omega^{3}t^{3}}{3!}+\cdots \}\\ & =e^{ijkh}e^{-i\omega t}\\ & =e^{i\left(jkh-\omega t\right)}, \end{array}$$

*which is the same solution obtained in [6].*

$$u\left(x,t\right)=e^{i\left(kx-\omega t\right)},\;x\in \mathbb{R},\;t>0,$$

*is the plane wave solution of the continuous form, where k is the wave number and* ω *denotes the frequency.*

**Figure 3:**
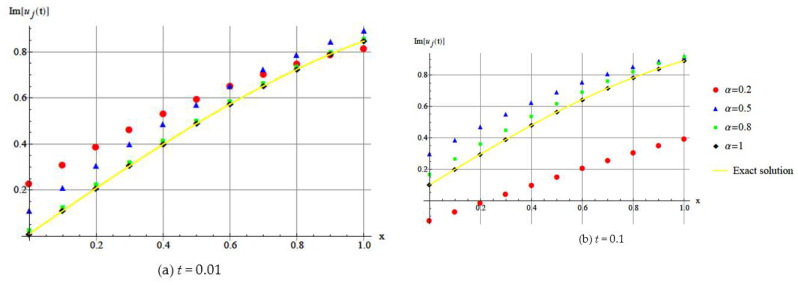
Numerical illustration of imaginary part of approximation solution *u*(*x*,*t*) by DHAM. (**a**) For *t* = 0.01; (**b**) For *t* = 0.1.

**Figure 4:**
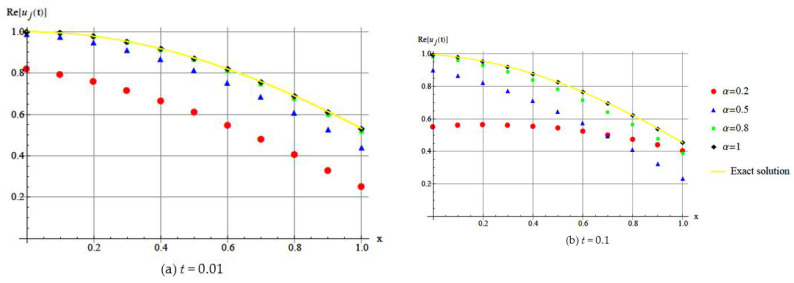
Numerical illustration of real part of approximation solution *u*(*x*,*t*) by DHAM. (**a**) For *t* = 0.01; (**b**) For *t* = 0.1.

Figure 3 and Figure 4 show the DHAM approximate solution of u(x,t) for different values of α, k=1 and q=2.

We can observe the different behaviors of the discrete fractional Schrödinger equations, with different fractional parameters.

**Figure 5:**
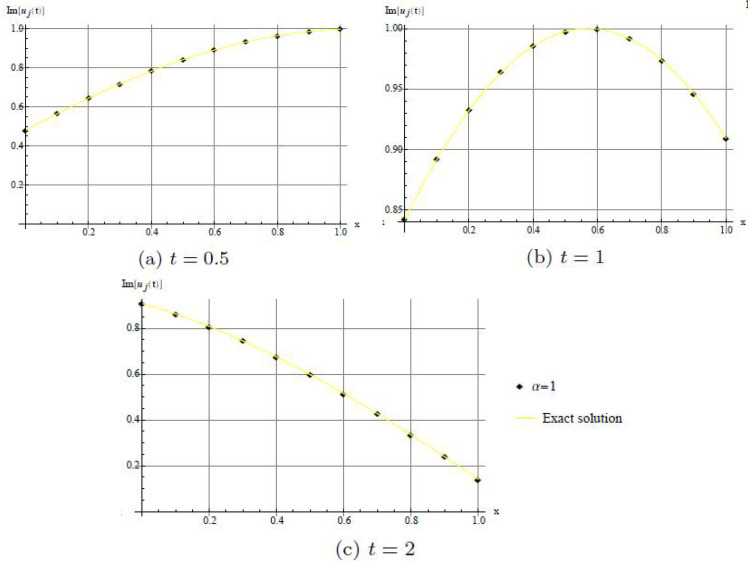
Comparison with numerical solution of *u*(*x*,*t*) by DHAM and the exact solution when α=1. (**a**) For *t* = 0.5; (**b**) For *t* = 1; (**c**) For *t* = 2.

In Figure 5, we show that the method has good agreement with imaginary part of the exact solution when α=1.

**Figure 6:**
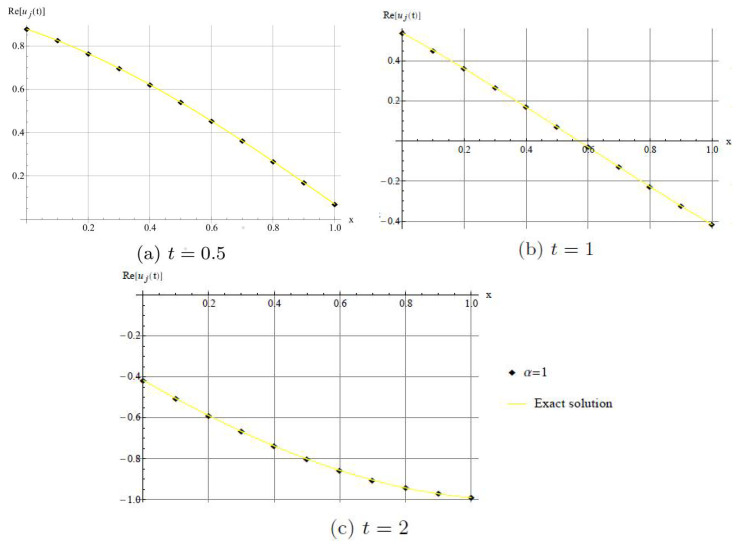
Comparison with numerical solution of *u*(*x*,*t*) by DHAM and the exact solution when α=1. (**a**) For *t* = 0.5; (**b**) For *t* = 1; (**c**) For *t* = 2.

In Figure 6, we show that the method has good agreement with real part of the exact solution when α=1.

**Example** **3.** *Consider time fractional space discrete nonlinear Burgers’ equation*

(27) $$D_{t}^{\alpha }u_{j}\left(t\right)+u_{j}\left(t\right)D_{h}u_{j}\left(t\right)=D_{h}^{2}u_{j}\left(t\right),\;j\in \mathbb{Z},\;t>0,\;0<\alpha \leq 1,$$

*with initial condition*

(28) $$u_{j}\left(0\right)=\sin jh.$$

*Initial value problem* (27) *and* (28) *is the discrete form of initial value problem for nonlinear fractional Burgers’ equation*

$$D_{t}^{\alpha }u\left(x,t\right)+u\left(x,t\right)u_{x}\left(x,t\right)=u_{xx}\left(x,t\right),\;t>0,\;x\in \mathbb{R},\;0<\alpha \leq 1,$$

*with initial condition*

u(x,0)=sinx.

*We take into consideration the linear operator:*

(29) $$L\left[\varphi_{j}\left(t;p\right)\right]=D_{t}^{\alpha }\left[\varphi_{j}\left(t;p\right)\right]=\frac{\partial^{\alpha }\varphi_{j}\left(t;p\right)}{\partial t^{\alpha }}$$

*with property* L[c]=0, *where c is constant. We can consider the nonlinear operator as*

(30) $$N\left[\varphi_{j}\left(t;p\right)\right]=D_{t}^{\alpha }\varphi_{j}\left(t;p\right)-D_{h}^{2}\varphi_{j}\left(t;p\right)+\varphi_{j}\left(t;p\right)D_{h}\varphi_{j}\left(t;p\right).$$

*Therefore, we construct the zeroth-order deformation equation by Equation* (2).

*For* p=0 *and* p=1 *, we can write*

$$\varphi_{j}\left(t;0\right)=u_{j,0}\left(t\right)=u_{j}\left(0\right),\;\varphi_{j}\left(t;1\right)=u_{j}\left(t\right),$$

*respectively. Thus, we obtain the mth-order deformation equation*

(31) $$L\left[u_{j,m}\left(t\right)-X_{m}u_{j,m-1}\left(t\right)\right]=\hbar H_{j}\left(t\right)R_{m}\left[{\vec{u}}_{m-1}\right],$$

*where*

(32) $$R_{m}\left[{\vec{u}}_{m-1}\right]=D_{t}^{\alpha }u_{j,m-1}\left(t\right)-D_{h}^{2}u_{j,m-1}\left(t\right)+\overset{m-1}{\underset{k=0}{\sum }}u_{j,k}\left(t\right)D_{h}u_{j,m-1-k}\left(t\right).$$

*For simplicity, we select again*$H_{j}\left(t\right)=1$*. So, the approximations of*$u_{j}\left(t\right)$*are only depend on auxiliary parameter*ℏ.

*The solution of the mth-order deformation equation for* m≥1 *give rise to*

(33) $$u_{j,m}\left(t\right)=X_{m}u_{j,m-1}\left(t\right)+\hbar J^{\alpha }R_{m}\left[{\vec{u}}_{m-1}\right].$$

*when we use the initial condition (28) along with (33), we attain the first three of terms of (33) as following:*

$$\begin{array}{cc} u_{j,0}\left(t\right) & =\sin jh\\ u_{j,1}\left(t\right) & =\left[\frac{\sin h}{2h}\sin2jh-\frac{2\left(\cos h-1\right)}{h^{2}}\sin jh\right]\frac{t^{\alpha }}{\Gamma \left(\alpha +1\right)}\hbar \\ u_{j,2}\left(t\right) & =\left[\frac{\sin h}{2h}\sin2jh-\frac{2\left(\cos h-1\right)}{h^{2}}\sin jh\right]\frac{t^{\alpha }}{\Gamma \left(\alpha +1\right)}\hbar \left(\hbar +1\right)\\ & +[\frac{\sin^{2}h}{2h^{2}}\sin2jh\cos jh+\frac{\sin h\sin2h}{2h^{2}}\sin jh\cos2jh\\ & -\frac{2\sin h\left(\cos h-1\right)\left(\cos h+2\right)}{h^{3}}\sin2jh+\frac{4{\left(\cos h-1\right)}^{2}}{h^{4}}\sin jh]\frac{t^{2\alpha }}{\Gamma \left(2\alpha +1\right)}\hbar^{2}\\ & \;\vdots \end{array}$$

*and so on.*

*Thus, we can conclude that*

$$u_{j}\left(t\right)=u_{j,0}\left(t\right)+u_{j,1}\left(t\right)+u_{j,2}\left(t\right)+\cdots$$

**Figure 7:**
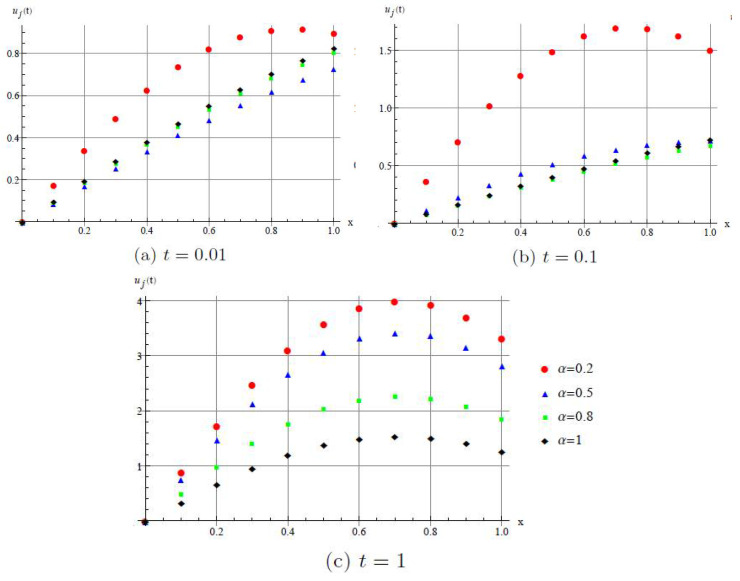
Numerical illustration of approximation solution *u*(*x*,*t*) by DHAM. (**a**) For *t* = 0.01; (**b**) For *t* = 0.1; (**c**) For *t* = 1.

Figure 7 shows the DHAM approximate solution of u(x,t) for different values of α.

We can see that the different behaviors of the discrete fractional Burgers’ equations for different fractional parameters.

## 4. Discussion and Conclusions

In this paper, the discrete HAM is successfully applied to find the solutions of linear and nonlinear fractional partial differential equations with time derivative α(0<α≤1). In contrast to all other analytic methods, it provides us with a simple way to adjust and convergence region of solution series by introducing an auxiliary parameter ℏ. This is an obvious advantage of the DHAM. We can simply choose the fractional operator $D_{t}^{\alpha }$ as the auxiliary linear operator. In this way, we obtained solutions in power series. Also we obtained the exact solutions in special case α=1, ℏ=−1 for some equations. However, it is well-known that a power series often has a small convergence radius. The results of test problems show that the DHAM is effective and reliable. It may also be a promising method to solve other nonlinear partial differential equations.
